# Supramolecular Organization of the Repetitive Backbone Unit of the *Streptococcus pneumoniae* Pilus

**DOI:** 10.1371/journal.pone.0010919

**Published:** 2010-06-15

**Authors:** Glen Spraggon, Eric Koesema, Maria Scarselli, Enrico Malito, Massimiliano Biagini, Nathalie Norais, Carla Emolo, Michèle Anne Barocchi, Fabiola Giusti, Markus Hilleringmann, Rino Rappuoli, Scott Lesley, Antonello Covacci, Vega Masignani, Ilaria Ferlenghi

**Affiliations:** 1 Genomics Institute of the Novartis Research Foundation, San Diego, California, United States of America; 2 Novartis Vaccines and Diagnostics, Siena, Italy; 3 Department of Evolutionary Biology, University of Siena, Siena, Italy; Technical University Munich, Germany

## Abstract

*Streptococcus pneumoniae*, like many other Gram-positive bacteria, assembles long filamentous pili on their surface through which they adhere to host cells. Pneumococcal pili are formed by a backbone, consisting of the repetition of the major component RrgB, and two accessory proteins (RrgA and RrgC). Here we reconstruct by transmission electron microscopy and single particle image reconstruction method the three dimensional arrangement of two neighbouring RrgB molecules, which represent the minimal repetitive structural domain of the native pilus. The crystal structure of the D2-D4 domains of RrgB was solved at 1.6 Å resolution. Rigid-body fitting of the X-ray coordinates into the electron density map enabled us to define the arrangement of the backbone subunits into the *S. pneumoniae* native pilus. The quantitative fitting provide evidence that the pneumococcal pilus consists uniquely of RrgB monomers assembled in a head-to-tail organization. The presence of short intra-subunit linker regions connecting neighbouring domains provides the molecular basis for the intrinsic pilus flexibility.

## Introduction

Most bacterial pathogens have long filamentous structures, known as pili or fimbriae, extending from their surface. These structures are often involved in the initial adhesion of the bacteria to host tissues during colonization. Over the past five decades, several distinct pilus types have been identified, most of which were described and characterized in Gram-negative bacteria.

Gram-negative pili are typically formed by non-covalent interactions between identical copies of pilin subunits, thus generating the pilus shaft. They have been reported to be involved in adherence to host cells, induction of cell signalling [1], transfer of genetic material [2], [3] and motility [4], [5]. In Gram-positive bacteria, these surface appendages were first detected in *Corynebacterium renale* by electron microscopy [6]. More recently, pili were reported in many diverse species such as *Actinomyces naeslundii, Corynebacterium diphtheriae,* and many pathogenic *Streptococcus spp* [7], [8], [9], [10]; [11]; [12]). During the past several years, pili of pathogenic streptococci have been under intense investigation, where they were shown to be fundamental for the adhesion/invasion process and pathogenesis [2], [13], [14], [15], [16].

Gram-positive pili differ from those of Gram-negative bacteria by the presence of covalently linked subunits containing a conserved LPXTG motif or a variant of it. This motif is the target of sortase enzymes which during pilus formation catalyse the covalent attachment of the consecutive backbone pilins by means of intermolecular isopeptide bonds formed between the Thr of the LPxTG motif and a Lys residue located at the N-terminus of the next subunit [17].

In *S. pneumoniae,* the genes coding for the pilus are contained in a 12 Kb pathogenicity island (the *rlrA* islet), consisting of seven genes of which *rrgA, rrgB,* and *rrgC* encode LPXTG-containing proteins [14], [18]. Only RrgB is strictly necessary for the pilus formation while the other two are ancillary proteins [2]. The major ancillary protein RrgA has been shown to be the pilus adhesin [19]. Consistently with its role in adhesion, recent data suggest that RrgA is located at the tip of the pilus, whereas the minor ancillary protein RrgC serves as the pilus anchor and is located at the base of the shaft [20], [56]).

For many years, no information on the structure and assembly of these pili were available. Novel awareness of the mechanism of Gram-positive pilus assembly arose from crystal structure determination of single pilus subunits of *S. agalactiae* (GBS) [21] and *S. pyogenes* (GAS) [22], however the determination of the macromolecular architecture of these important structures still remained elusive.

Here we present for the first time the three dimensional structure of the *S. pneumoniae* native pilus obtained using a combination of electron microscopy (EM) and single-particle image reconstruction method. The rigid body fitting of the RrgBD2-D4 X-ray coordinates into the pilus electron density map highlights a head-to-tail arrangement made exclusively by the RrgB protein. These results provide insights into the molecular structure of the repetitive backbone unit of the pilus as well as on the regions that are exposed on its surface and may be important for the development of therapeutic inhibitory molecules and for the next generation of protein vaccines.

## Results

### Microscopy and image analysis of native pneumococcal TIGR4 pili

**Figure 1:**
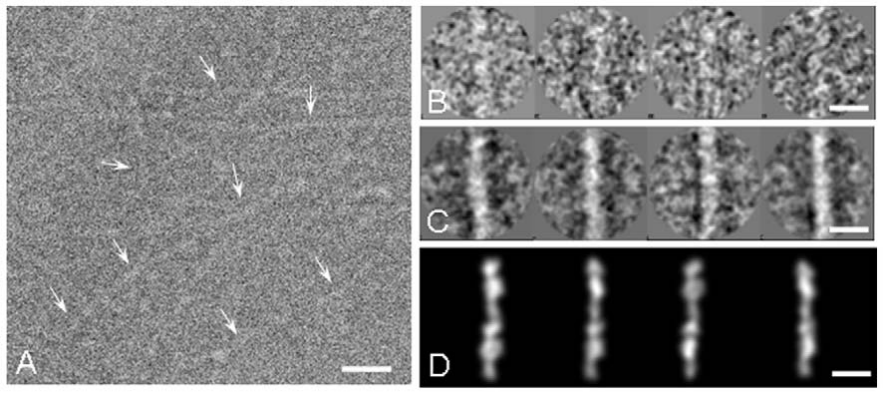
Negative stain EM of purified *S. pneumoniae* pili. (A) The micrograph shows a network of elongated thin structures indicated by white arrows. (B) Set of raw images of purified *S. pneumoniae* pili. (C) Representative class averages of purified *S. pneumoniae* pili segments. (D) Re-projection of the 3D reconstruction of the pilus. Scale bar in panel A 20 nm, scale bar in panels B, C and D 10 nm.

To shed light on the molecular architecture of native pili we examined the 3D architecture of *S. pneumoniae* pili by applying the single-particle reconstruction method to low-dose transmission electron microscopy (TEM) images of negative stained native pili. When observed by TEM purified *S. pneumoniae* TIGR4 pili preparation appeared as long and thin filaments. Random presence of pili aggregated into bundles of various diameters or twines were also observed. On TEM micrographs (Figure 1A, arrowheads) *S. pneumoniae* pili appeared as faint, gently curved filaments with a variable length ranging from 100 nm up to 1000 nm and with an average width of 5 nm. High magnification TEM images revealed pili as elongated structures with a “pearl necklace” organization

To understand pilus morphology at molecular level over 5000 independent segments of linear fibres were manually selected from digitized micrographs and boxed into a 164×164 pixel box in order to generate the three-dimensional reconstruction of the pilus. The final structure was solved at 22 Å resolution applying the single-particle approach [23].

**Figure 2:**
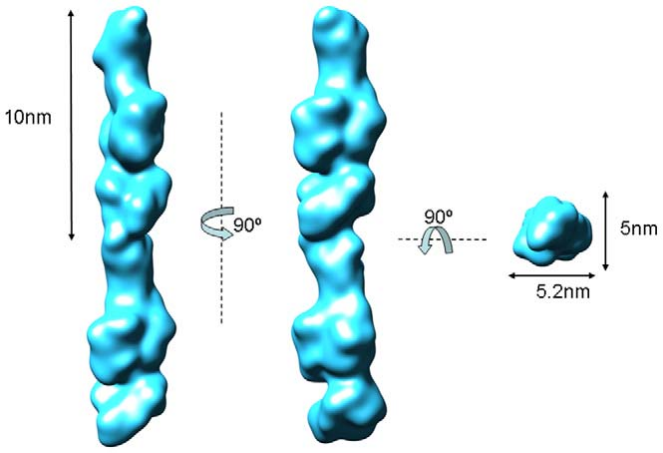
Three-dimensional reconstruction of the *S. pneumoniae* pilus. Surface representation of the *S. pneumoniae* pilus viewed along three orthogonal directions. The reconstruction is rendered at the molecular mass expected for two RrgB subunits (66 kD each). Structure is viewed along three orthogonal directions. Surface representation rendered with Chimera (Pettersen EF et al 2004).

Boxed segments were pre-aligned to a future-less reference cylinder centred into the image box (Figure 1B). The correctly pre-aligned segments were then subjected to multivariate statistical analysis (MSA) [23], [24] for classification. The resulting class-averages determined by angular reconstruction applying a C1 point-group symmetry [25] showed *S. pneumoniae* pili as fibres with an irregular “pearl necklace” appearance. Visual inspection allowed us to observe that most of the class averages displayed pili composed by individual elongated beads organized in a head-to-tail arrangement (Figure 1C). Depending on the orientation a lateral concavity could be detected in some of the beads, giving thus them a “bean” appearance. Since the concavity was not always detectable along the pilus this suggested that the beads did not follow a defined helical rule of assembly. In order to confirm the data a final set of class averages were compared to re-projections of the 3D reconstruction obtained (Figure 1D). The electron density map of the reconstructed full-length RrgB scaffold (Figure 2) resulted in an extremely compact structure clearly showing a contiguous organization of individual subunits, with each single subunit made by a thin connecting region (∼3 nm width) followed by larger (∼5.2 nm width) and smaller (∼5 nm width) globular densities separated by a lateral concavity (Figure 2). The filament interior is tightly-packed. A twist along the longitudinal axis was observed between two neighbouring subunits along the length of the pilus. Different degrees of twist ranging between 17° and 22° were measured. The refinement of the pilus structure was performed using a 3D model of the fibre composed of 2 averaged subunits. The projections around the long axis of the averaged pilus were used for multi-reference alignment (MRA) [26] of the different pilus segments. Two EM reconstructions of the pilus were generated, which differed in the number of filament segments used, the defocus range, and the approach to contrast transfer function (CTF) correction. Comparison of these two reconstructions by Fourier shell correlation method [27] (FSC, threshold 0.50) provided a resolution estimated 22 Å.

### Structure of major pneumococcal pilin RrgB fragment

**Figure 3:**
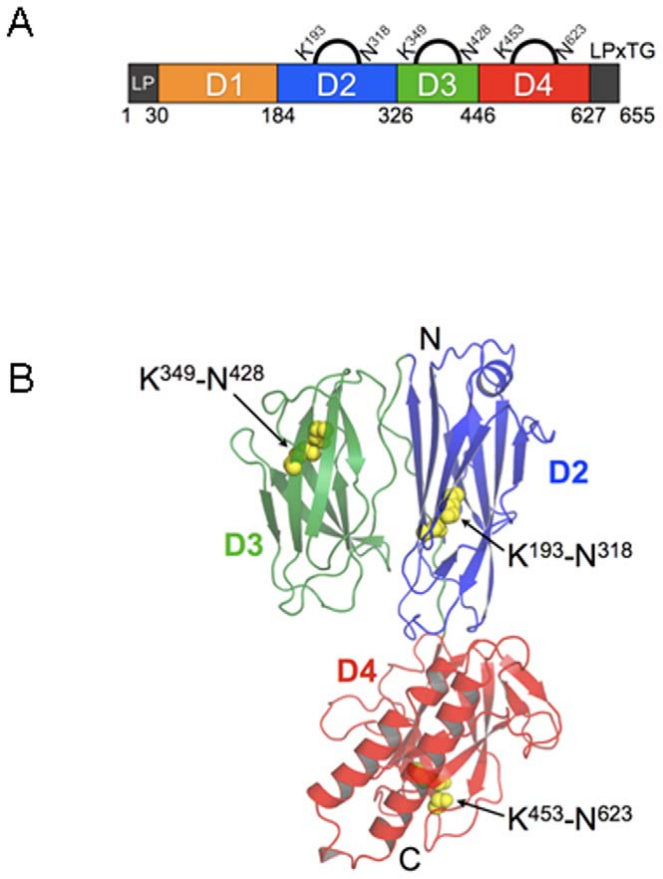
Structure of RrgB. A) Sequence organization of the full-length RrgB protein. D1 is depicted in yellow, while D2, D3 and D4 domains are respectively in blue, green and red. Position of the intra-molecular isopeptide bonds, the leader peptide (LP) and LPXTG motif are indicated. B) Ribbon Diagram showing the three domains of RrgB coloured as in A. Isopeptide bonds in each domain are shown as yellow spheres. Figure produced with Pymol (http://www.pymol.org/).

Attempts to obtain full-length RrgB crystals were made with no success. Crystallisable construct of fragments of RrgB were derived from limited proteolysis and mass spectroscopy experiments (see Materials and Methods construct design for details). The fragment of RrgB designated RrgBD2-D4 (residues 184–627, Figure 3A) was crystallized in three different crystal forms (Table 1) (in space groups P2_1_2_1_2_1_, P6_1_22 and C222_1_). All the three forms of RrgBD2–D4 possess identical tertiary structure and all exhibited root mean square deviations (rmsds) with each other on aligned carbon alpha atoms of less than 7 Å (Table S1). Due to this, reference to the RrgBD2–D4 will be made to the orthorhombic crystal structure only, the coordinate set used in the analysis.

**Table 1:** Crystal forms of RrgBD2–D4 and of RrgBD2–D3 fragments.

	RrgB-184-627	RrgB-184-627	RrgB-184-627	RrgB-187-448
**Data collection**				
PDB ID	2X9W	2X9X	2X9Y	2X9Z
Spacegroup	P2_1_2_1_2_1_	P6_1_22	C222_1_	C2
No. Mol. in ASU	1	1	1	1
Unit Cell (Å) a/b/c	67.36/74.06/104.46	142.56/142.56/89.40	43.67/157.75/147.67	94.97/46.63/64.38 β = 115.48
Wavelength (Å)	0.9795	0.9795	1.54	0.9795
Resolution (Å)	50-1.9	50-1.5	50.0-2.3	50.0-1.3
Total Reflections unique,(no observations)	40672 (529986)	85582 (812884)	21514 (135832)	61593 (192302)
Completeness % (highest shell)	99.4 (89.3)	99.7 (98.3)	96.3 (86.3)	98.0 (90.7)
*R*_merge_, % (highest shell)	0.071 (0.93)	0.062 (0.91)	0.2 (0.705)	0.09 (0.71)
Highest Res. Shell, (Å)	1.95-1.92	1.55-1.5	2.38-2.34	1.32-1.3
Mean I/σ (I), (highest shell)	26.0 (1.4)	35.9(1.28)	8.7 (1.3)	24.6 (1.0)
Mean Redundancy (highest shell)	13.0 (5.8)	9.5 (4.5)	6.3 (4.1)	3.3 (1.8)
**Phasing**				
Mean Figure of merit for resolution range (Å)	0.2 (50.0 2.3)	-	-	-
**Refinement**				
No. refs. working set	70612	85061	21265	56542
No. refs. test set	3579	4253	1092	2866
*R*_cryst_ (*R*_free_ *)†	0.17 (0.23)	0.168 (0.2)	0.2 (0.25)	0.15 (0.17)
Rmsd bonds, Å	0.012	0.004	0.004	0.005
Rmsd angles, °	1.294	0.849	0.764	1.033
Average B, Å^2^	29.9	20	35.3	16.2
Maximum Likelihood based on *R*_free_, Å^‡^	0.25	0.23	0.67	0.16
**Ramachandran Plot**				
Most Favored, %	90.9	91.6	90.6	90.4
Additional Allowed, %	8.8	8.1	9.2	9.6
Generously Allowed, %	0.3	0.3	0.3	0.0
Disallowed, %	0.0	0.0	0.0	0.0

†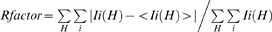 where I_i_ is the scaled intensity of the ith measurement, and <I_i_> is the mean intensity for that reflection.

*R_free_  =  as for R_factor_, but for 5.0% of the total reflections chosen at random and omitted from refinement.

Each RrgBD2–D4 crystal form contained one molecule in the asymmetric unit, consisting of an elongated polypeptide (84 Å in length, 50 Å in width) made of three immunoglobulin-like domains (named D2, D3 and D4).

A further crystal form designated RrgBD2–D3 (residues 141–592) was also derived via the construct design process. Crystals grown from these constructs belong to the monoclinic (space group C2) crystal form and diffracted to high resolution (Table 1) but lacked any ordered electron density outside regions defining domains D2 and D3. Because of the close packing of the molecules in this crystal form, it seems very likely that crystal growth occurred after a degradation event during the crystallization process.

The first 262 residues (184–446) form a dual domain (D2 and D3) made of two β-sandwiches of approximately 100 residues present in all crystal forms. The C-terminal 180 residues (446–627) form a third domain (D4) that contains an additional anti-parallel α-helical motif. As all domains described consist of modified bacterial immunoglobulin domains, β-strands are named according to the standard greek key nomenclature, represented in Figure S1.

All three domains D2, D3 and D4 are structurally homologous to each other, consisting of a pair of four-stranded β-sheets forming a β-sandwich configuration. Despite the topological equivalence, considerable variation exists between the domains and superposition of the three domains D2/D3, D2/D4 and D3/D4 results in the hydrophobic core being aligned with rmsd of 3.6, 3.3 and 2.8 Å on 68, 56 and 71 Cα coordinates, respectively. The C-terminal β-sheet of domain D3 contributes one additional strand to domain D2 completing the 4-stranded β-sheet of the β-sandwich. Further details on the structural similarity between D2 and D3 domains are reported in Text S1. This intercalation of secondary structural elements contributes to the stability of the D2–D3 fragment. The helical component in domain D2, one 3–10 helix and a short 6 residue stretch (aa 283–289), is consistent with that found in the eukaryotic IgG immunoglobulin fold. Domains D2 and D3 are oriented together in such a way that the six loops form a flat relatively broad surface of >5000 Å^2^ (Figure 3).

The core of these domains is made of seven β-strands within two β-sheets arranged in a greek key motif reminiscent of the immunoglobulin domain fold (Figure 3B). This core, which corresponds to the collagen binding domain (Cna_B), is present in several surface proteins of Gram-positive bacteria, including the backbone protein Spy0128 of *S. pyogenes* (PDB code 3B2M) [22] and the minor ancillary pilus protein GBS052 of *S. agalactiae* (PDB code 2PZ4) [21]. The tertiary structure of the RrgB fragment relative to these two proteins is also similar, with the exception that the RrgB fragment contains an additional domain (D3) arranged laterally relative to the other two structures, which only contain two longitudinally oriented Ig-like domains.

**Figure 4:**
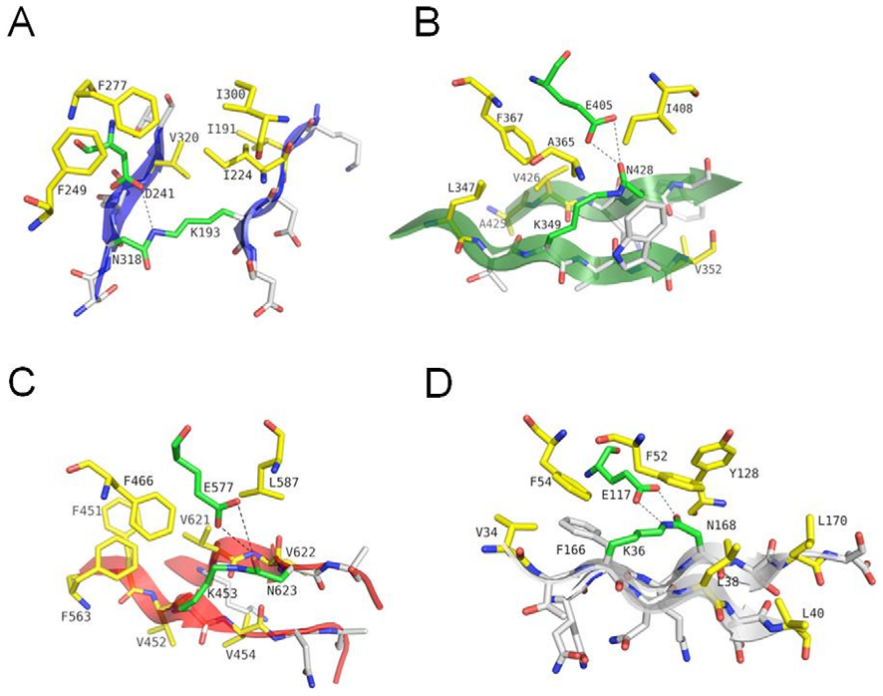
Isopeptide Bonds of RrgB and Spy0128. A) and B) the isopeptide bond between residues Lys193 and Asn318 in domain 2 (D2) of RrgB. Dashed lines show the distance of the carboxylate anion provided by Asp241; the isopeptide bond between Lys 349 and Asn428 in domain 3 (D3) and the position of Glu405 contributing the carboxylate anion; the isopeptide bond between Lys 453 and Asn623 and the position of Glu577 in domain 4 (D4). C) Sigma-a weighted *2Fo*–*Fc* map contoured at 1σ level is shown for the isopeptide bond in D4 of RrgB. D) Isopeptide bond between Lys36 and Asn168 in Spy0128 (drawn from PDB 3b2m). Carbon atoms are coloured green for residues involved in the formation of the isopeptide bonds, yellow for neighbouring hydrophobic residues, and gray for residues adjacent to the isopeptide bond. Oxygen and nitrogen atoms are coloured red and blue, respectively. Figure produced by Pymol.

As first shown by Kang *et al.* [22], each domain of the backbone subunit spy0128 of *S. pyogenes* contains a stabilizing Asn-Lys isopeptide bond within its core and an acidic residue in its proximity, which provides the carboxylate anion needed for the formation of the isopeptide link. We also find that each of the three crystallized domains of RrgB contains intra-molecular isopeptide bonds involving: Asn 318-Lys193 for D2; Asn 428-Lys349 for D3, and Asn 623-Lys453 for D4 (Figure 4A, B, and C). The isopeptide bonds in domains D3 and D4 are localized in the proximity of Glu residues (aa 405 and 577, respectively) as observed for Spy0128 (Figure 4D), while the isopeptide bond of the D2 domain is close to Asp241. Comparison of the area surrounding the isopeptide bonds of RrgB with those of GBS052 and Spy0128 shows that they are relatively well conserved, with the exception of RrgB D2 domain where Asp241 is positioned in a reverse orientation with respect to the Glutamate residues present in the other two. In RrgB the carboxylate group is located in closest proximity to the ε-amino of the Lys rather than to the carboxyl-oxygen of the Asn (Figure 4A). Despite these differences, the amino acids surrounding the carboxylate anions of the catalytic residues are largely conserved, each constituting a hydrophobic cavity nearby the carboxylate contributing residue. In domain D2, Asp241 is surrounded by Phe277, Phe249, Ile300, Ile224 and Val230; in domain D3 Glu405 is surrounded by Val426, Phe367, Ala365 and Ile408; finally in domain D4 Glu577 is surrounded by Leu587, Phe466, Phe563, Phe451 and Ala464 (Figure 4A, B and C).

The presence and nature of the isopeptide bonds in D2, D3 and D4 were also supported by mass spectrometry data (Texts S2 and S3 and Figure S2)

### Gram positive pili contain different types of backbone subunits

**Figure 5:**
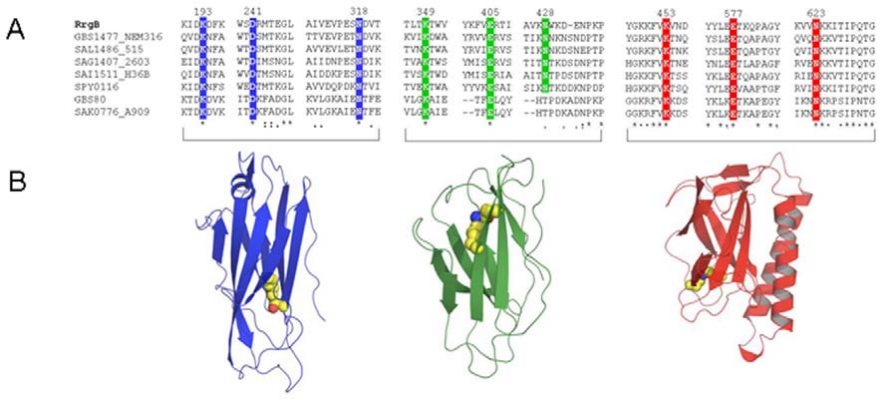
Sequence alignments of related pili proteins from Gram-positive bacteria. A) Sequence alignment showing intra-molecular isopeptide bond formation which involves conserved residues Lysine (K), Aspartic Acid (D), Glutamic Acid (E) and Asparagine (N). Residues are highlighted and coloured according to the domain localization. UniProtKB/TrEMBL accession codes are shown; RrgB-TIGR4, RrgB-6BSP and RrgB-23FTW represent the sequences of the three RrgB alleles of *S. pneumoniae*; SAG0645 (also known as GBS80) belongs to PI-1 of *Streptococcus agalactiae*; GBS1477, SAL1486, SAG1407 and SAI1511 belong to GBS PI-2a, whereas SAK0776 belongs to GBS PI-2b; Spy0116 belongs to the M4 serotype of *Streptococcus pyogenes*. The sequence alignment was obtained and edited by Jalview/ClustalW [55]. B) Cartoon representation of RrgB single domains coloured as in 1A) is shown on the bottom. Yellow spheres depict the residues forming the isopeptide bonds.

Sequence comparison was performed between RrgB and pilus backbone proteins of other streptococcal pili. Significant similarity resulted between RrgB and the backbone proteins of *S. pyogenes* and *S. agalactiae* (Figure 5). In particular RrgB is more similar to backbone subunits of *S. agalactiae* pilus islands 1 and 2a [28] and to serotype M4 *S. pyogenes* backbone protein Spy0116 [11], where the level of sequence conservation ranges from 36 to 44% of sequence identity. With only one exception, the residues involved in the formation of the intra-molecular isopeptide bonds present in D2, D3 and D4 are also conserved in backbone proteins belonging to pilus island 1 and 2a of GBS and in M4 GAS Spy0116 (Figure 5A), indicating that these subunits might also share a similar global folding with RrgB. In contrast, the overall similarity between RrgB and the pilus backbone of pilus island 2b of GBS and most GAS pilus backbone proteins (including the crystallized spy0128) revealed only a very limited sequence conservation (data not shown). In line with this, while RrgB consists of four independently folded domains, spy0128 has only two distinct domains and is much shorter in length (665aa of RrgB versus 340 aa of spy0128). These diversities suggest that Gram-positive pili can adopt a similar overall architecture despite using different types of molecules as major building blocks.

### Fitting of the RrgB D2-D4 crystal coordinates into the pilus density map

**Figure 6:**
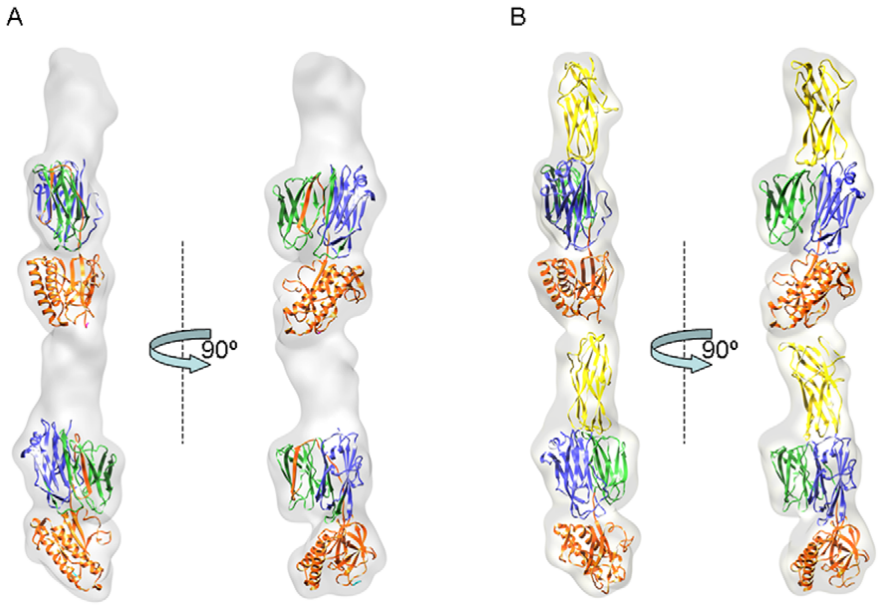
Fitting of the RrgBD2–D4 crystal structure and of the RrgBD1 computer model into the 3D map of the *S. pneumoniae* pilus. A) Semi transparent rendered surface representations of the 3-D map of the *S. pneumoniae* pilus viewed along the z-axis tilted by 90° showing the docking of two copies of the atomic resolution RrgBD2–D4 subunit into the pilus reconstruction. Empty densities present above the two fitted RrgBD2–D4 copies have a volume of ∼21×e^3^ Å^3^ each. B) Same semi-transparent rendered surface representations as in panel A showing the docking into the pilus reconstruction of two copies of the RrgBD2–D4 subunit and contemporaneously of two computer model RrgBD1 domains. The docking reveals that the inter-subunit density (7 Å thick) present between the upper and lower subunits could accommodate the 8-residue C- terminal tail, not present in the RrgBD2–D4 crystal. Crystal structures are in cartoon representation and the three domains are coloured following the nomenclature of Figure 3. Figure produced with Pymol (http://www.pymol.org/). Surface representation and molecule rendered with Chimera [29].

In order to investigate subunit arrangement and interactions in the *S. pneumoniae* pilus, a rigid-body fitting of two RrgBD2–D4 crystal fragments into the electron density map was performed by using CHIMERA [29] (Figure 6A). The C-terminal immunoglobulin domain (D4) of the crystal structure matched well into the smaller globular density present below the groove with the core of seven β-strands placed internally the filament and the additional anti-parallel α-helical motif exposed on the surface. The fitting confirmed that pilus volume (∼155×e^3^ Å^3^) and dimensions (52 Å in width and 252 Å in length) could accommodate two RrgBD2–D4 molecules organized in a head-to-tail arrangement with rotations ranging between 17° and 22° along the vertical axis of the lower RrgBD2–D4 subunit in respect to the upper one. Here both the flattened surface of the D2–D3 dual domain and the D4 domain could interface with the D1 domain of the neighbouring subunit. Furthermore the fitting suggested that the inter-subunit density (7 Å thick) could accommodate the 8-residue C-terminal tail not present in the RrgB crystal. Notably, no information about the neighbouring subunits or the missing residues in the truncated pilin model was introduced into the fitting at any stage. Therefore, the packing of the 84 Å long molecule within the 60 Å diameter EM density without significant collision independently validates the EM reconstruction and docking procedures.

Moreover, the rigid body fitting performed with the electron density map countered at the same threshold levels that corresponded to the molecular mass of two RrgB subunits (∼132 kD total mass) showed that the two RrgBD2–D4 crystal structures occupied a total volume of ∼117×e^3^ Å^3^ leaving two regions of unoccupied volume (∼21×e^3^ Å^3^) in the density map. The two remaining extra unoccupied volumes, both present above the N-terminus of each fitted RrgBD2–D4 crystal fragment (Figure 6A), easily accommodated the volume of two computer modelled D1 domains (RrgBD1) (Text S4, Figure S3A), each containing 156 residues. Each D1 computer model was first fitted manually using CHIMERA by placing as much of the atomic structure as possible fully into the EM density map, approximately in the position thought to be correct. This step was then followed by a rigid body fitting using CHIMERA and optimized, as previously, for the spatial frequency band of 22–60 Å (Figure 6B). The low correlation values (<0.55) and the absence of clashes were both indications of the correct orientation of the D1 computer models into the 3D pilus density in respect to the RrgBD2–D4 crystal fragment orientation (Figure S3B). Finally, the packing of the RrgB subunits within the EM density was the only one validating the EM reconstruction. Any attempt of fitting done with the computer models of the two ancillary proteins at the spatial frequency band of 22–60 Å did not satisfy the spatial restrains and generated a too high level of collisions.

## Discussion

Fibrillar structures have been recently found in Gram-positive bacteria complementing the wide range of Gram-negative pathogens that since long have been known to express pili on their surface. Pili were first observed in the Gram-positive species *Corynebacterium renale* by electron microscopy, than followed by their detection on the surface of other Gram-positive bacteria such as *Corynebacterium diphtheriae*, *Streptococcus salivarius* and *Streptococcus sanguis.* Recently these elongated appendages have been found on the surface of the principal streptococcal pathogens including Group A *streptococci*, Group B *streptococci* and *S. pneumoniae*. In addition to colonization and adhesion, Gram-positive pili have also been associated to other functions among which biofilm formation and immune evasion [15], [17], [30]. Pilus subunits are immunogenic in humans [13] and able to elicit a protective response when tested in mouse models of infection [12], [31]. Pilus expression increases pathogenicity in animal models [32], [33], and enhances adhesion to epithelial cells [14], [34], [35].

To accurately define the structure and assembly mechanism of the pneumococcal pilus we determined both a low-dose EM reconstruction of the pilus filament and a high resolution crystal structure of the backbone subunit.

Here the crystal structure of the RrgBD2–D4 backbone subunit fitted into the EM reconstruction of the *S. pneumoniae* pilus reveals for the first time the polymeric architecture of a Gram-positive pilus indicating a head-to-tail organization of individual backbone subunits. The measured unoccupied volume present between two neighbouring subunits is compatible with the predicted density of the flexible 8-residue C-terminus sequence ^624^KKVTIPQT^631^ that is missing from the crystal structure. This region contains Thr631, implicated in the formation of the inter-molecular isopeptide bond that links the C-terminus of one subunit to the N-terminal region of the next one in the row. Sequence analysis of RrgB indicates the presence of two lysines potentially implicated in the isopeptide bond formation: i) Lys183 which is part of a canonical pilin motif (^180^VYPKN^184^); ii) Lys162 which can be nicely aligned with Lys161 of Spy0128, previously identified by Kang *et al*. [22]. When mapped onto the predicted model of the RrgBD1 domain, both residues were well exposed and located on the same face of the molecule. However, according to the model, only Lys162 could be close enough to the presumed position of the LPXTG motif of the neighbouring RrgB molecule to be involved in the formation of an inter-molecular isopeptide bond. Nevertheless, experimental evidence is still needed to discriminate the essential Lys.

The analysis of the rigid body fitting indicates that not only the surface-surface interaction between neighbouring subunits but also the presence of the inter-molecular isopeptide bond constrains the flexibility of the pilus. The limited curviness observed in the pili could be conferred by the hinge region of 2–4 residues which links D1 to D2 in each individual subunit. This internal flexibility is suggested also by the proteolysis experiments where the presence of a proteolytic cleavage site between D1 and D2 indicates that a mobile loop connects the two domains.

Another important aspect of pilus biogenesis is the understanding of how the ancillary proteins are incorporated into the pilus backbone. Originally, two distinct mechanisms were hypothesized. The first assumed that the ancillary proteins are incorporated in the pilus shaft in a similar manner as the backbone subunits, either interspersed between the backbone subunits or located at the extremities of the fiber. The second one sustains that the ancillary proteins are associated laterally to the pilus shaft generating a branched structure.

Previous reports [36] [19] [37] [20] showed by Immunogold EM that RrgA and RrgC were distributed in clusters along pili when organized in bundles. The single pilus structure presented in this work and in Hilleringmann *et al.* [56] clearly show that the pilus shaft is made by multiple copies of RrgB organized in a head-to-tail linear structure and with the two opposite tips decorated by the two ancillary proteins. Thus the presence of the ancillary proteins clusters observed along the bundles could be a consequence of the disposition of single pili along the bundle. Recent works on GBS and *C. diphtheriae* have suggested that the minor pilin may anchor the pili on the cell wall [30] [38], whereas another recent paper shows that in GAS the major ancillary protein is only attached at the tip of the fiber, consistently with its role in adhesion [39]. These data are in agreement with our observations that in *S. pneumoniae* the ancillary proteins are not appended laterally, conferring to the pilus a pearl on a string appearance of identical subunits bound to each other. Moreover rigid-body fitting clearly indicates that the pilus density can correspond only to a linear assembly of RrgB monomers, excluding that other molecules, apart from RrgB, are incorporated into the pilus shaft or appended laterally. Therefore, as in the case of *C. diphtheriae* the most probable scenario for the pilus of *S. pneumoniae* is the one that contemplate the presence of the major ancillary protein RrgA at the distal tip where it could be more available for adhesion, whereas the RrgB backbone provides the structure with the elasticity required to reach the host cell receptors.

The work described above shows the powerful synergy and mechanistic insights that can result from a combined EM, X-ray crystallography and Mass Spectrometry approach. The three-dimensional structure of the pilus generated from TEM images fitted with high resolution crystal structure of the major fragment of RrgB have provided a detailed molecular view of the backbone of *S. pneumoniae* pilus, and could be a key-model for the study of the assembly, attachment and function of the pili in Gram-positive bacteria.

## Materials and Methods

### Bacterial strains and culture conditions

*S. pneumoniae* type 4 strain TIGR4 was employed [40]. The pneumococcal strains were stored at –80°C in 12% glycerol and routinely grown at 37°C in 5% CO_2_ on Tryptic Soy Agar (Becton Dickinson) supplemented with 5% defibrinated sheep blood or in Tryptic Soy Broth (Becton Dickinson). When appropriate, erythromycin and kanamycin (Sigma-Aldrich) as selection marker were used.

### Native TIGR4 pili purification

*S. pneumoniae* TIGR4 strain was chosen as starting material as the bacteria belong to a clinical relevant serotype 4 isolate, the sequence of which is known [40].

Native pili of TIGR4 and TIGR4Δ*rrgA* were purified essentially according a protocol described by Hilleringmann *et al*. [37]. Purified pili fractions were judged to be homogeneous based on electron microscopy and SDS-PAGE. Samples were stored at –80°C or liquid nitrogen until further use.

### Electron microscopy

A 5 µl aliquot of purified pili preparation with a final concentration of 0.052 µg/ µl was applied to 200-square mesh copper grids coated with a thin carbon film and let stand for 5 min. Excess of solution was blotted by Whatman filter paper. The grids were first washed by streaming several drops of PBS over the grids. They were subsequently negatively stained by two drops of 1% buffered PTA (pH 6.5). The last drop was left on the grids for 17 s. Finally the grids were washed with several drops of ddH_2_O, the excess of liquid was soaked off by Whatman filter paper and air dried. The grids were observed using a CM200 FEG Philips Electron Microscope (FEI, Eindhoven, The Netherlands), equipped with a GATAN GIF 2002 post column energy filter (Gatan, Pleasanton, California, United States). All images were collected at an accelerating voltage of 200 kV and a nominal magnification of 50000×, on Kodak SO163 film. Micrographs where checked for astigmatism and drift on an optical diffractometer prior to digitisation.

### Image processing

Micrographs taken at 50000× of magnification were digitized on an IMACON 949 scanner at spacing of 7.95 µm resulting in a nominal sampling of 1.6 Å/pixel-1. Analysis of defocus and Contrast Transfer Function (CTF) using the Medical Research Council (MRC) program CTFFIND3 [41] and IMAGIC 5 [26] showed that the first zero corresponds to ∼17–19 Å. Since only a moderate resolution of the 3-D reconstruction of the *S. pneumoniae* pilus was required in order to identify the arrangement of the backbone subunits, the final 3-D map was obtained at 22 Å resolution using the 0.5 threshold of the Fourier shell correlation (FSC) [27]. Pili segments were picked manually from digitized images using the command “helixboxer” from the software EMAN [42]. Digitized pili images were cut into individual repeats by using boxes of 164×164 pixels, with overlapping ends, using 10 pixel shift for each box, so that adjacent boxes had 90% overlap. Images were band-pass filtered at 17–200 Å to remove background and normalized. The individual pili segments were treated as single particles. In a first analysis, the segments were selected and pre-aligned interactively, subsequently the pre-aligned repeats were aligned using alignments with only limited angular ranges (−5°, +5°), finally a vertical alignment has been performed using as a future-less reference the projection of a model cylinder, with a 5 nm width that corresponds to the width of the pilus measured in the images, followed by translational alignment perpendicular to the cylinder axis only. All the aligned and filtered images were consistent: they all presented centred rods with similar diameters. The only major differences were the surrounding stain distributions. Aligned pili segments were than classified by MSA to sort images into class averages with similar features. The class averages obtained have an improved signal-to-noise ratio and represent characteristic molecular views of the pilus. Most class averages showed pili with subunit-like features. Several iterations of alignments and MSA classification led to homogeneous class averages showing pili with globular subunits arranged linearly. The initial model was determined from four side views of the pilus and one end view [43]. In a first approximation, the end view was taken as rotationally symmetrised average. The 3-D map was than refined by adding class averages of the side views and a reprojection along the z-axis as the end view. Re-projections of the final 3-D were compared for consistency with input class averages to check the accuracy of the Euler angles assigned [23]. Image processing of the pilus was performed using software IMAGIC-5 [26]. The final 3-D map of the *S. pneumoniae* pilus was refined at 22 Å resolution (FSC = 0.5) [27] by iterating procedures of alignment and classification. 3D rendered surface representations were visualized in UCSF Chimera [29].

### Construct Design

Full length RrgB (aa1-665) was expressed and purified in *E.coli* strain HK100, according to standard protocols [44]. The resultant protein was purified by Ni-NTA IMAC (Quiagen) and eluted by a HEPES elution buffer, Buffer A (20 mM HEPES pH 7.3, 150 mM NaCl, 1 mM TCEP). 80 µl TPCK-Trypsin (Pierce) was washed three times with 400 µl Buffer A, and then resuspended in 800 µl of the same buffer. 25 µl of the TPCK-Trypsin suspension was added to 50 µl protein (0.5–1.5 mg/ml) and incubated at 37°C, 250 rpm for 4 hours. The immobilized trypsin was removed by centrifugation and the proteolyzed samples were then submitted for LCMS. Mass spec data were analyzed by PAWS (Genomic Solutions Inc.) to determine possible truncation boundaries. The resultant constructs were cloned in the pSpeedET vector by the PIPE cloning method [45]. Positive clones were verified by DNA sequence analysis, and expressed in an identical way to the full length construct [46].In all, ten constructs were generated via this methodology with boundaries, 24–227, 52–460, 109–562, 139–590,141–592, 184–627,163–615,191–337 and 281–484 of the full length RrgB construct.

### Crystallization

All crystallization experiments were carried out in 96 well low profile Greiner crystallization plates in a nanodroplet sitting drop vapour diffusion format with 480 conditions screens performed at both 4 and 20° [46]. Equal volumes of protein concentrated to 10 mg/ml were added to the reservoir solutions to create a total drop volume of 500 nl. Three crystal forms of the RrgBD2–D4 (containing residues 184–627) constructs were produced belonging to spacegroups P2_1_2_1_2_1_, P6_1_22 and C222_1_ in conditions 0.05 potassium dihydrogen phosphate, 20% PEG-8000 pH 4.5 4°C, 1.0 M Sodium Citrate 0.1 M Imidazole pH 8.0 at 20°C and 0.2 M Lithium Sulfate, 30% Peg-4000 0.1 M Tris pH 8.5 4°C respectively. The other constructs which ultimately produced a two domain version of the structures RrgBD2–D3, (containing residues 141–592 of full length RrgB) were produced with identical crystallization screens in crystal condition 30% PEG-6000 0.1 M Citrate pH 5.0 at 20°C. All crystals were mounted using 20% glycerol as a cryo-protectant prior to cooling to 100°K for data collection.

### Data Collection and Structure Solution

Data were collected at beamline 5.0.2 and 5.0.3 of the ALS and were processed with the HKL2000 package [47] . Data collection for phasing was performed on the orthorhombic crystal form of RrgB184–627 at the Selenium edge with an inverse beam strategy. Substructure solution, phasing, density modification and initial model building was performed with SOLVE and RESOLVE [48], [49] on the primitive orthorhombic crystal form. Given that only one Seleno-Methionine residue existed in the 441 residues present in the asymmetric unit, the anomalous signal was relatively weak (ΔF/σΔF∼1.2 on all data between 50 and 2.3 Å) but implementation of a brute force searching strategy over various resolution ranges and redundancies resulted in the location of the substructure and initial phases, capable of building the model. Subsequent refinement and building was performed with Phenix and Coot [50], [51]. All other crystallographic manipulations were carried out with the CCP4 package [52]. Solutions of all other crystal forms were performed by molecular replacement using the orthorhombic crystal form as a search model and Phaser [53] followed by refinement and building with Phenix and Coot [50], [54]. The geometry of all structures is excellent and all residues are in allowed regions of the Ramachandran plot (Table 1).

### Fitting of the X-ray coordinates into the electron density map

The fitting was carried out independently for two individual RrgBD2–D4 crystals and optimized for the spatial frequency band of 22–60 Å. The correlation values between the fitted atomic structures of two copies of RrgBD2–D4 and the 3-D map corresponding to the upper and lower subunits of the pilus reconstruction were both 0.66. All other orientations of two subunits into the 3-D map resulted in lower correlation values (<0.5 for each single subunit). Moreover, the alternative checked orientations did not satisfy the spatial restrains on the distance between neighbouring subunits. Alternative fittings of two adjacent subunits had tilts and rotations that increased the distance between the N-terminus of one fragment and the C-terminus of the next one. Basically the first two modified immunoglobulin domains D2 and D3 of the crystal structure aligned well with the larger globular density present in the pilus reconstruction, placing the pair of four-stranded β-sheets parallel to the filament axis and facing outward.

## Supporting Information

Figure S1 Structure of RrgB. A) Ribbon Diagram showing the three domains of RrgB coloured as in Figure 3B. Intra-isopeptide bonds in each domain are shown as yellow spheres. Figure produced with Pymol (http://www.pymol.org/). Greek-key representation of the secondary structure organization of RrgB D2-D4 domains (panel B) and the prototypic immunoglobulin fold (panel C).
(3.08 MB TIF)

Figure S2 Peptide mass fingerprinting of RrgB Domain 4. Each signal is labelled with an m/z ratio and the amino acid position of the corresponding tryptic peptide. A) Signals labelled in red are consistent with peptides linked by an isopeptide bound between Lys453 and Asp623. The signal at m/z 1675.94 is consistent with one trypsin missed cleavage while the signal at m/z 1290.74 is consistent with no missed cleavage. Signal labelled with an asterisk is consistent with the tryptic N-terminal and C-terminal peptide of the cloned D4 domain of sequence MASVTYGK (m/z 856.50) and ITLEHHHHHH (m/z 1297.64), respectively. Signal labelled with a T is generated by trypsin autolysis products. B) MS/MS spectrum of parental ion at m/z 1675.94. Only b and y series are reported. A scheme of the fragmentation is shown at the top of the figure.
(3.34 MB TIF)

Figure S3 Fitting of the RrgBD1 computer model into the 3D map of the *S. pneumoniae* pilus. A) Computer model of the D1 domain and sequence alignment of RrgB D1 to the N-terminal domain of Spy0128 (PDB code 3B2M). Gold rectangles depict the localization of beta strands on the crystal structure. B) The overall protein fold is represented as a ribbon; the side-chains of Lys41 and Asn184, involved in the intra-molecular isopeptide bond are highlighted. Lys162, with putative involvement in the inter-molecular isopeptide bond is evidenced in grey. B) After fitting, D1 and D2-D4 coordinates were merged into a single file and overlapping atoms were removed. The resulting RrgBD1-D4 model was visually inspected for absence of steric conflicts and minimized with the same protocol used for D1. Threading was performed with SwissPDBViewer, surface representation and molecule rendered with Chimera. Crystal structures are in cartoon representation and the three domains are coloured following the nomenclature of Figure 3. Figure produced with Pymol (http://www.pymol.org/). Surface representation and molecule rendered with Chimera.
(10.23 MB TIF)

Table S1 Root mean square deviations (rmsd) of the three crystal forms of RrgBD2-D4.
(0.03 MB DOC)

Text S1 Structural relationship between D2 and D3 domains. Definition of the reciprocal orientation of domains D2 and D3.
(0.03 MB DOC)

Text S2 Presence of intra-molecular isopeptide bonds in D2, D3 and D4 are confirmed by MS. Details on the characterization of isopeptide bonds.
(0.03 MB DOC)

Text S3 In-gel digestion and mass spectrometric analysis. Details on mass spectroscopy analysis.
(0.03 MB DOC)

Text S4 Computer modelling of the RrgBD1 domain. Details on the generation of the D1 computer model.
(0.03 MB DOC)
