# Supplementary figures and images for: Supramolecular Organization of the Repetitive Backbone Unit of the Streptococcus pneumoniae Pilus
Source: PLoS One. 2010 Jun 15;5(6):e10919. doi: 10.1371/journal.pone.0010919 (PMC2886109; doi:10.1371/journal.pone.0010919)

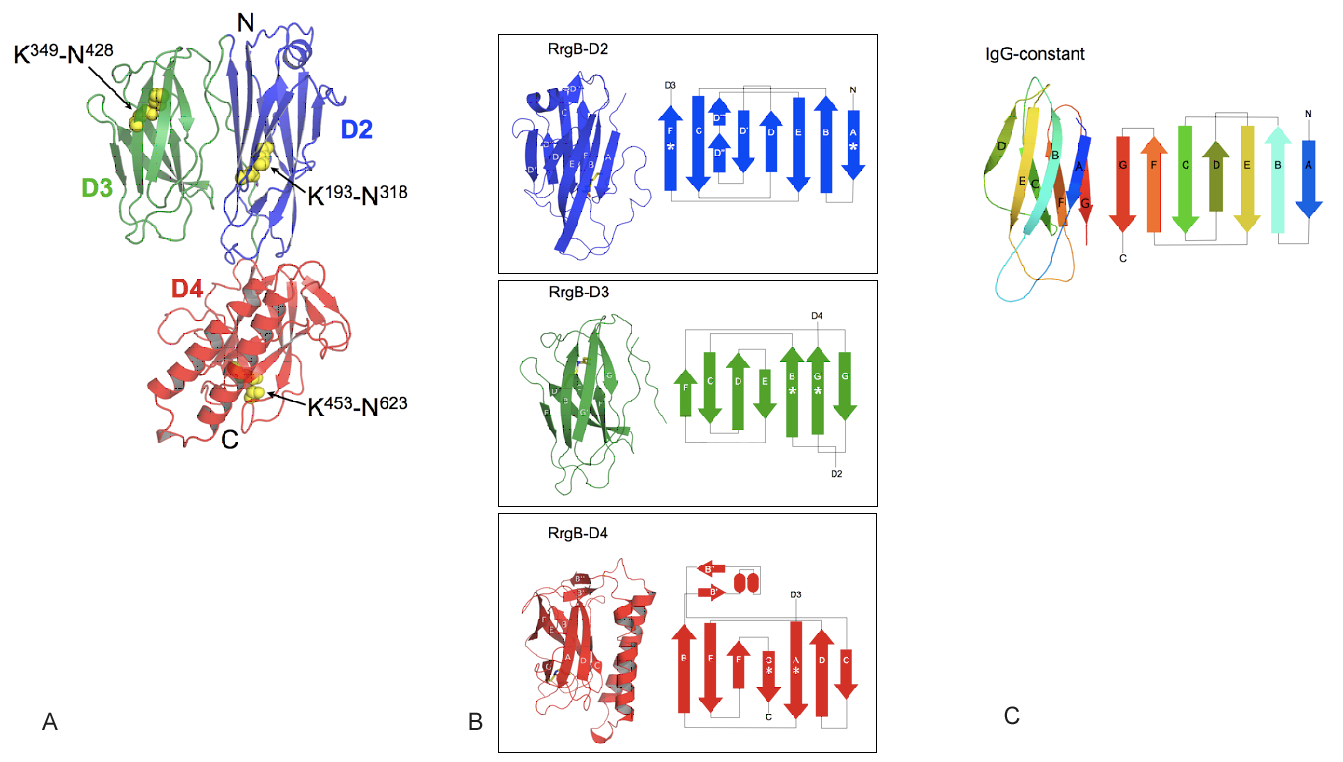

Supplement: Figure S1 — Structure of RrgB. A) Ribbon Diagram showing the three domains of RrgB coloured as in Figure 3B. Intra-isopeptide bonds in each domain are shown as yellow spheres. Figure produced with Pymol (http://www.pymol.org/). Greek-key representation of the secondary structure organization of RrgB D2-D4 domains (panel B) and the prototypic immunoglobulin fold (panel C). (3.08 MB TIF) [file pone.0010919.s001.tif]

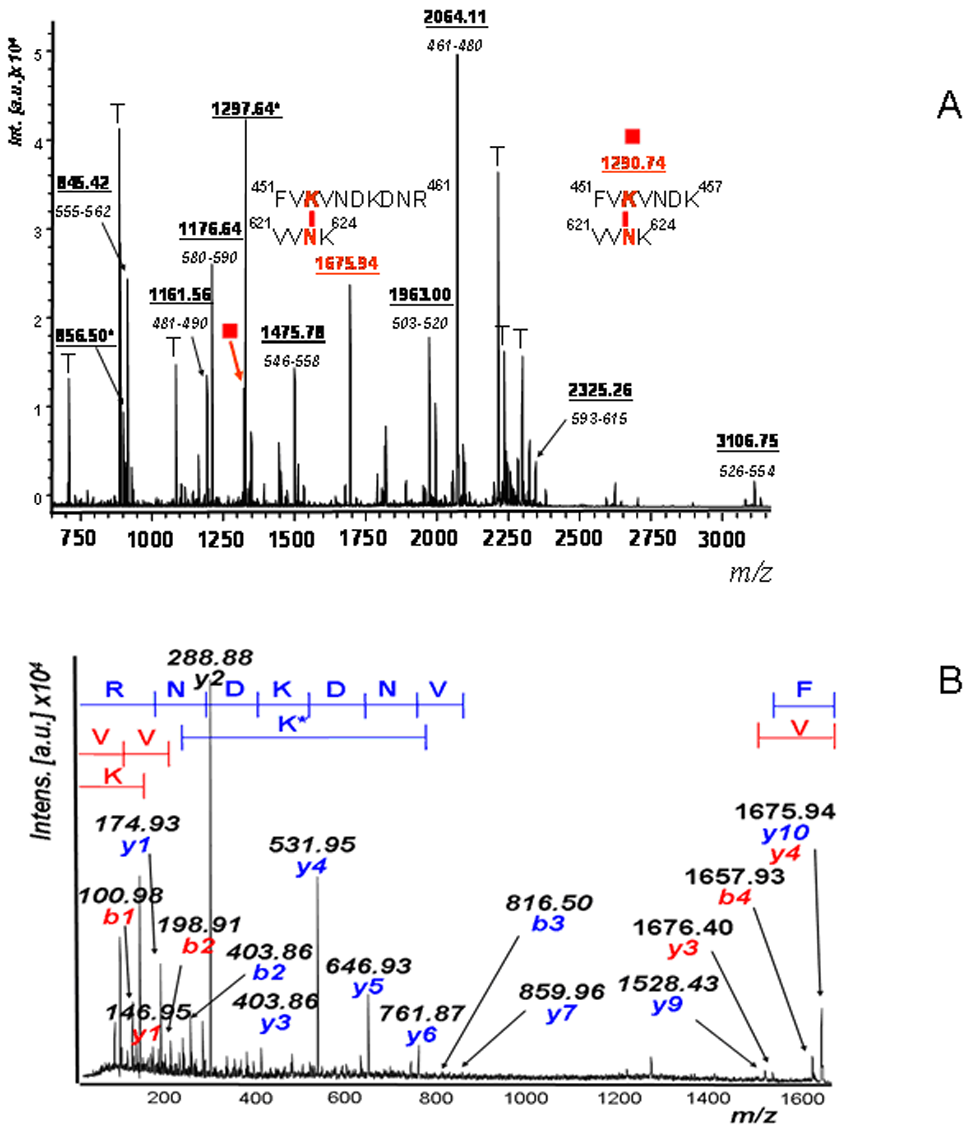

Supplement: Figure S2 — Peptide mass fingerprinting of RrgB Domain 4. Each signal is labelled with an m/z ratio and the amino acid position of the corresponding tryptic peptide. A) Signals labelled in red are consistent with peptides linked by an isopeptide bound between Lys453 and Asp623. The signal at m/z 1675.94 is consistent with one trypsin missed cleavage while the signal at m/z 1290.74 is consistent with no missed cleavage. Signal labelled with an asterisk is consistent with the tryptic N-terminal and C-terminal peptide of the cloned D4 domain of sequence MASVTYGK (m/z 856.50) and ITLEHHHHHH (m/z 1297.64), respectively. Signal labelled with a T is generated by trypsin autolysis products. B) MS/MS spectrum of parental ion at m/z 1675.94. Only b and y series are reported. A scheme of the fragmentation is shown at the top of the figure. (3.34 MB TIF) [file pone.0010919.s002.tif]

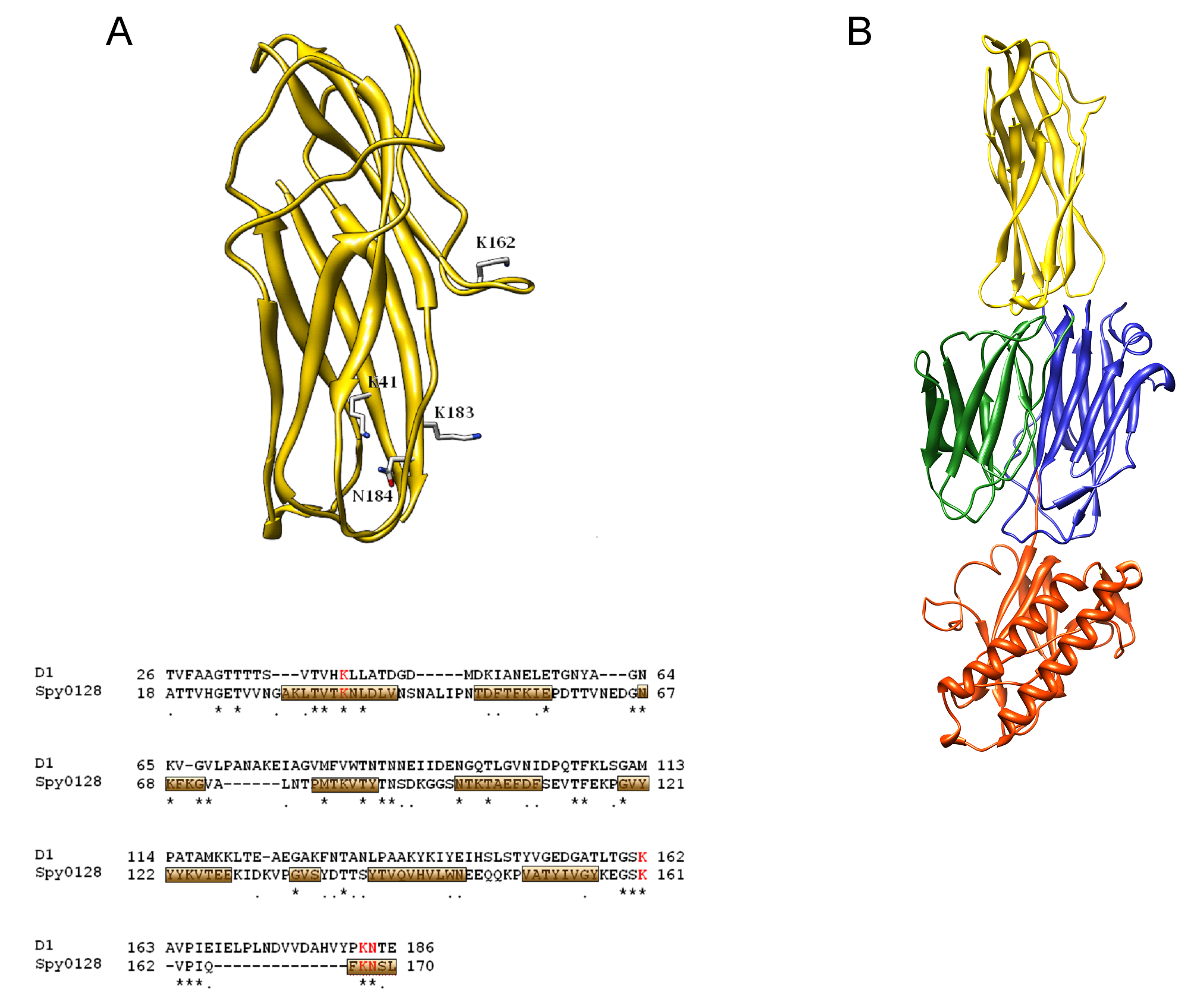

Supplement: Figure S3 — Fitting of the RrgBD1 computer model into the 3D map of the S. pneumoniae pilus. A) Computer model of the D1 domain and sequence alignment of RrgB D1 to the N-terminal domain of Spy0128 (PDB code 3B2M). Gold rectangles depict the localization of beta strands on the crystal structure. B) The overall protein fold is represented as a ribbon; the side-chains of Lys41 and Asn184, involved in the intra-molecular isopeptide bond are highlighted. Lys162, with putative involvement in the inter-molecular isopeptide bond is evidenced in grey. B) After fitting, D1 and D2-D4 coordinates were merged into a single file and overlapping atoms were removed. The resulting RrgBD1-D4 model was visually inspected for absence of steric conflicts and minimized with the same protocol used for D1. Threading was performed with SwissPDBViewer, surface representation and molecule rendered with Chimera. Crystal structures are in cartoon representation and the three domains are coloured following the nomenclature of Figure 3. Figure produced with Pymol (http://www.pymol.org/). Surface representation and molecule rendered with Chimera. (10.23 MB TIF) [file pone.0010919.s003.tif]
