# Supplementary material for: Supramolecular Organization of the Repetitive Backbone Unit of the Streptococcus pneumoniae Pilus
Source: PLoS One. 2010 Jun 15;5(6):e10919. doi: 10.1371/journal.pone.0010919 (PMC2886109; doi:10.1371/journal.pone.0010919)
Supplement: Table S1 — Root mean square deviations (rmsd) of the three crystal forms of RrgBD2-D4. (0.03 MB DOC) [file pone.0010919.s004.doc]

Table S1

|  | **2w9w** | **2w9x** | **2w9y** |
| --- | --- | --- | --- |
| **2w9x** | 0.67/428* | -- | -- |
| **2w9y** | 0.50/439 | 0.50/428 | -- |
| **2w9z** | 0.59/262 | 0.52/262 | 0.51/262 |

* rmsd/number of aligned residues
