# Supplementary material for: Supramolecular Organization of the Repetitive Backbone Unit of the Streptococcus pneumoniae Pilus
Source: PLoS One. 2010 Jun 15;5(6):e10919. doi: 10.1371/journal.pone.0010919 (PMC2886109; doi:10.1371/journal.pone.0010919)
Supplement: Text S1 — Structural relationship between D2 and D3 domains. Definition of the reciprocal orientation of domains D2 and D3. (0.03 MB DOC) [file pone.0010919.s005.doc]

S1

**Structural relationship between D2 and D3 domains**

Although domains D2 and D3 were topologically equivalent, superposition with Secondary-Structure Matching (SSM) produced a root mean square deviation (rmsd) of 3.7Å over 68 aligned Cα residues. The C-terminal β-sheet of domain D3 contributed by one additional strand to domain D2 completing the four-stranded β-sheet of the β-sandwich. This intercalation of secondary structural elements contributes to the stability of the D2-D3 fragment. The helical component in domain D2, with a single 3-10 helix and a short 6 residue stretch (residues 283-289), was consistent with the Ig-like immunoglobulin fold. The two domains were oriented in such a way that the six loops formed a flat relatively broad surface > 5000Å^2^.
