# Supplementary material for: Supramolecular Organization of the Repetitive Backbone Unit of the Streptococcus pneumoniae Pilus
Source: PLoS One. 2010 Jun 15;5(6):e10919. doi: 10.1371/journal.pone.0010919 (PMC2886109; doi:10.1371/journal.pone.0010919)
Supplement: Text S2 — Presence of intra-molecular isopeptide bonds in D2, D3 and D4 are confirmed by MS. Details on the characterization of isopeptide bonds. (0.03 MB DOC) [file pone.0010919.s006.doc]

S2

**Presence of intra-molecular isopeptide bonds in D2, D3 and D4 are confirmed by MS.**

The presence of intra-molecular isopeptide bonds was investigated by Mass Spectrometry. Domain D4 (residues 446-627) was cloned as a His-tag polypeptide, expressed in *E. coli*, and purified on a Nickel affinity chromatography. The presence of intra-molecular isopeptide bonds was investigated by an in gel tryptic digestion of the D4 domain. The resulting peptide fragments were analyzed by MALDI TOF mass spectrometry (shown as a mass fingerprint in Figure S2A). Ten peaks were assigned to the D4 domain, resulting in 60.2% sequence coverage over the domain. Of the twelve total peaks two masses were not assigned to theoretical tryptic peptides, relating to signals *m/z* 1290.74 and *m/z* 1675.94. These two values were consistent with the molecular mass of the peptide ^621^VVNK^624^ linked by an isopeptide bond to either the peptide ^451^FVKVNDK^457^ (expected *m/z* 1290.73) or to the peptide ^451^FVKVNDKDNR^461^ (expected *m/z* 1675.90), which resulted from the partial trypsin digestion (Figure S2A). The unbound peptide fragment peaks ^621^VVNK^624^ and ^451^FVKVNDK^457^ were not observed indicating that the two fragments were linked by an isopeptide bond. To confirm the sequence of the two fragments involved in the isopeptide bond formation (^451^FVKVNDKDNR^461^) the ion of m/z 1675.94 was fragmented by MALDI TOF/TOF mass spectrometry and the primary sequence deduced from the MS/MS spectrum was in agreement with the sequence of the cross-linked peptides (Figure S2B). Similar analyses were applied to domains D2 and D3. Also in these cases, mass spectrometry was able to confirm the presence of intra-molecular isopeptide bonds (data not shown).
