# Supplementary material for: Supramolecular Organization of the Repetitive Backbone Unit of the Streptococcus pneumoniae Pilus
Source: PLoS One. 2010 Jun 15;5(6):e10919. doi: 10.1371/journal.pone.0010919 (PMC2886109; doi:10.1371/journal.pone.0010919)
Supplement: Text S3 — In-gel digestion and mass spectrometric analysis. Details on mass spectroscopy analysis. (0.03 MB DOC) [file pone.0010919.s007.doc]

S3

**In-gel digestion and mass spectrometric analysis**

Colloidal Coomassie Blue G-250 stained bands were excised from SDS–polyacrylamide gel, destained and *in-gel* digested in 5mM NH_4_HCO_3_ with 12 µg/ml of modified trypsin (Trypsin, Promega, Madison, USA) for a night at 37°C. Peptide solution was directly spotted on a matrix PAC target (Prespotted AnchorChip 96, set for Proteomics, Bruker Daltonics). Dried spots were washed with 0.6 µl of a solution of 70% (vol/vol) ethanol, 0.1% (vol/vol) TFA. Peptide mass spectra were recorded with a MALDI-TOF/TOF mass spectrometer UltraFlex (Bruker Daltonics, Bremen, Germany). Ions generated by laser desorption at 337 nm (N_2_ laser) were recorded at an acceleration of 25 kV in the reflector mode. In general, about 200 single spectra were accumulated for improving the signal/noise ration and analyzed by FlexAnalysis (version 2.4, Bruker Daltonics). External calibration was performed using standard peptides pre-spotted on the target.

MS/MS experiments were performed under CID conditions with the LIFT cell voltage parameters set at 19.0 kV for a final acceleration of 25 kV (reflector voltage) and a pressure in the LIFT cell around 10^−7^ mbar. The precursor ion selector was set manually to the first monoisotopic peak of the molecular ion pattern. MS/MS spectra were acquired from 1000 laser shots by adjusting for each peptide the laser intensity above the threshold for generation of molecular ions. Peptide identification and sequence assignment were carried out using BioTools and SequenceEditor (versions 3.0, Bruker Daltonics).
