# Supplementary material for: Supramolecular Organization of the Repetitive Backbone Unit of the Streptococcus pneumoniae Pilus
Source: PLoS One. 2010 Jun 15;5(6):e10919. doi: 10.1371/journal.pone.0010919 (PMC2886109; doi:10.1371/journal.pone.0010919)
Supplement: Text S4 — Computer modelling of the RrgBD1 domain. Details on the generation of the D1 computer model. (0.03 MB DOC) [file pone.0010919.s008.doc]

S4

**Computer modelling of the RrgBD1 domain**

Based on the assumption that D1 domain of RrgB and the N-terminal domain of spy0128 share similar overall topology, a homology model of D1 was constructed. The sequence alignment program ClustalW was used to align the sequences of D1 (residues 25-185) and spy0128 (residues 34-186). D1 was threaded onto the X-ray coordinates of the template (PDB code 3B2M) with SwissPDB Viewer (<http://www.expasy.org/spdbv/>), 2). Manual intervention was used to refine regions corresponding to gaps in the alignment. The resulting model was refined by energy minimization with the Gromos force field. The quality of the model was checked using PROCHECK. The Ramachandran plot showed that 99.2 % of the residues in the model were located in allowed regions. Despite D1 and spy0128 had only 18% of identical residues, the final D1 model met some criteria of acceptable overall quality such as proper torsion angles, expected location of proline residues within the Ramachandran plot and burying of most hydrophobic side chains. Although the distance between Lys41 and Asn184 could suggest a possible engagement of their side chains into an intra-molecular isopeptide bond, no Glu/Asp residues could be identified in proximity of these residues. Furthermore, the hydrophobic cavity surrounding the intra-molecular isopeptide bonds of D2, D3 and D4 was not observed in the D1 model. Accordingly, mass spectroscopy failed to unambiguously identify residues involved in a possible bond (data not shown). For all these reasons, the presence of an intra-molecular isopeptide bond in the D1 domain remained elusive. To model the full-length RrgB, Glu186 of D1 was superimposed to the corresponding residues of the D2-D4 structure. After fitting, D1 and D2-D4 coordinates were merged into a single file and overlapping atoms were removed. The resulting model (Figure S3B) was visually inspected for absence of steric conflicts and minimized with the same protocol used for D1.
